# RNF6 promotes the migration and invasion of breast cancer by promoting the ubiquitination and degradation of MST1

**DOI:** 10.3892/etm.2022.11255

**Published:** 2022-03-14

**Authors:** Yajuan Huang, Yufeng Zou, Zhigang Jie

Exp Ther Med 23:118, 2022; DOI: 10.3892/etm.2021.11041

Subsequently to the publication of the above article, the authors have realized that errors were made during the compilation of Fig. 3 on p. 6. Specifically, an incorrect set of images were used for the Transwell migration and invasion assay experiments shown in Fig. 3C.

The authors have re-examined their raw data and identified the data that should have been included in this figure. The revised version of Fig. 3 is shown below, now including the correct data for Fig. 3C and re-quantification of the data in Fig. 3D and E. Note that this error did not have a major impact on either the overall results or on the conclusions reported in this study. The authors are grateful to the Editor of *Experimental and Therapeutic Medicine* for allowing them the opportunity to publish this corrigendum. All the authors agree to the publication of this corrigendum, and apologize to the readership for any inconvenience caused.

## Figures and Tables

**Figure 3:**
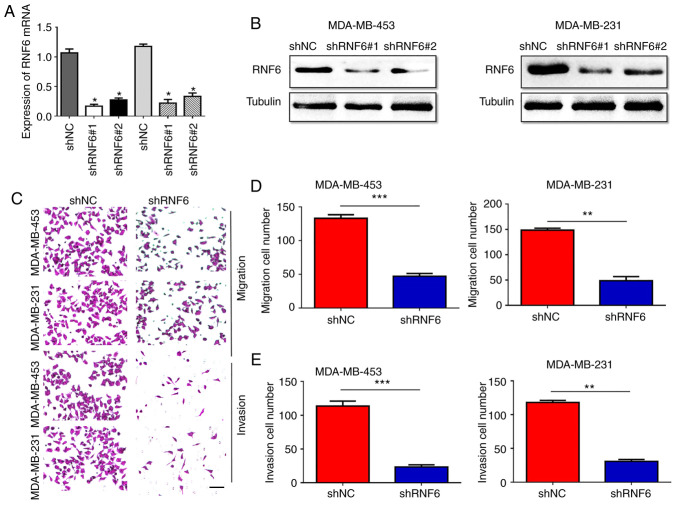
RNF6 knockdown inhibits breast cancer metastasis and invasion *in vitro* and *in vivo*. (A) Reverse transcription-quantitative PCR and (B) western blotting were performed to detect RNF6 mRNA and protein expression in MDA-MB-453 and MDA-MB-231 cells stably transfected with shNC or shRNF6. ^*^P<0.05 vs. shNC. (C) Microscopy images of Transwell (D) migration and (E) invasion assays of MDA-MB-453 and MDA-MB-231 cells with stable RNF6 knockdown, Scale bar, 50 µm ^**^P<0.01; ^***^P<0.001. Magnification, x100. RNF6, ring finger protein 6; sh, short hairpin; NC, negative control.

